# Fuzzy Pattern Tree Evolution Using Grammatical Evolution

**DOI:** 10.1007/s42979-022-01258-y

**Published:** 2022-08-06

**Authors:** Aidan Murphy, Muhammad Sarmad Ali, Douglas Mota Dias, Jorge Amaral, Enrique Naredo, Conor Ryan

**Affiliations:** 1 Lero and University of Limerick, Limerick, Ireland; 2 Rio de Janeiro State University, Rio de Janeiro, Brazil

**Keywords:** Grammatical evolution, Pattern trees, Fuzzy logic

## Abstract

A novel approach to induce Fuzzy Pattern Trees using Grammatical Evolution is presented in this paper. This new method, called Fuzzy Grammatical Evolution, is applied to a set of benchmark classification problems. Experimental results show that Fuzzy Grammatical Evolution attains similar and oftentimes better results when compared with state-of-the-art Fuzzy Pattern Tree composing methods, namely Fuzzy Pattern Trees evolved using Cartesian Genetic Programming, on a set of benchmark problems. We show that, although Cartesian Genetic Programming produces smaller trees, Fuzzy Grammatical Evolution produces better performing trees. Fuzzy Grammatical Evolution also benefits from a reduction in the number of necessary user-selectable parameters, while Cartesian Genetic Programming requires the selection of three crucial graph parameters before each experiment. To address the issue of bloat, an additional version of Fuzzy Grammatical Evolution using parsimony pressure was tested. The experimental results show that Fuzzy Grammatical Evolution with this extension routinely finds smaller trees than those using Cartesian Genetic Programming without any compromise in performance. To improve the performance of Fuzzy Grammatical Evolution, various ensemble methods were investigated. Boosting was seen to find the best individuals on half the benchmarks investigated.

## Introduction

Machine learning (ML) has been very successful in finding solutions to a vast swathe of real-world problems and contributed to innovation in products and research. Since the turn of the millennium, the number of applications of ML has increased owing to the availability of vast collections of data which can be cheaply stored and massively parallel computer power, new powerful training algorithms, the emergence of new hardware platforms based on graphics cards with GPUs, and the wide availability of open-source libraries [10]. Such environments provide ML systems with the ability to solve complicated real-world problems and routinely achieve new state-of-the-art results. Remarkably, it is seen in image classification and some other areas that ML systems have surpassed human performance [17].

The success of ML and learning algorithms in general, although wildly successful in terms of results and predictions, have their shortcomings. The most forceful is the lack of transparency, which identifies the so-called black-box models. In these black-box models, it is very demanding or even unfeasible to recognize how the ML system makes its decision or to extract the knowledge of how the decision is made. Put simply, it does not permit a human being, expert or not, to examine, comprehend, and make sense of how the model reaches its conclusions.

To try to solve these questions, Explainable Artificial Intelligence (XAI) [1, 5] has appeared, concerned with the interpretability of state-of-the-art ML. The main purpose of this field of research is to design a set of models and interpretable methods that are more explainable than the state of the art. This is all done while retaining the high levels of predictive performance which have been achieved [7].

Fuzzy logic and fuzzy set theory have supplied a framework in which it is possible to generate interpretable models [8, 18]. It allows the knowledge obtained from the data to be communicated in a comprehensible form to humans, close to natural language. This gives any model which uses fuzzy sets a high degree of interpretability [20]. Most developed fuzzy models are rule-based fuzzy systems (FBRS) that can represent both classification and regression functions and for which there are many strategies developed for the synthesis of these models [8]. Deriving fuzzy models based on easily interpretable rules is not an easy task. Depending on the application, many rules may be necessary, with many antecedents, that make understanding the model a troublesome task. However, a system which contains relatively few rules can be easily interpreted. Its predictive accuracy may be compromised by this restriction, though.

This is an extended version of a paper published in the proceedings of the 12th International Conference on Evolutionary Theory and Applications [25]. The previous work is built upon by investigating the effects various ensemble methods have on the performance of the classifiers. The techniques considered were aggregation, adaptive boosting, and gradient boosting. A method based on the theory of fuzzy sets, Fuzzy Pattern Trees (FPT), is used. An FPT is not based on rules, but on a hierarchical method. The FPT is now learned with a well-known method, Grammatical Evolution (GE).

GE is flexible enough to derive feasible models such as FPTs. It can easily address different problem types by augmenting the grammar and the evaluation function. Therefore, GE can find FPT models which solve classification tasks and achieve explainability simultaneously. This combination of GE, Fuzzy Logic, and a hierarchical structure gives a valuable opportunity to address the new research lines in XAI. Experimental results show that FPTs evolved using GE solve benchmark classification problems with competitive results against state-of-the-art methods and find superior results in three of them.

“Background” discusses the main background concepts. These include FPTs, Cartesian GP (CGP), and GE. “Fuzzy GE” describes the proposal and details the contributions of this work. Next, “Experimental Setup” presents the experimental setup, outlining all of the considered variants and performance measures. “Results” presents and discusses the main experimental results of the research. Finally, “Conclusions” presents the conclusions and puts forward the future work derived from this research.

## Background

### Fuzzy Sets and Fuzzy Pattern Trees

**Fig. 1:**
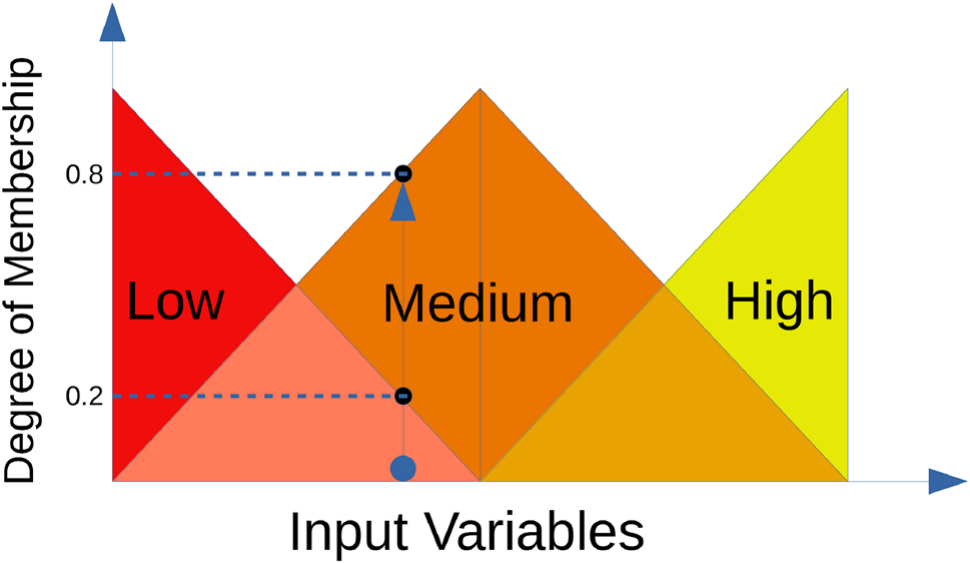
Fuzzy system

Fuzzy sets are an extension of regular sets, which were first introduced by [44]. They differ from traditional “crisp” sets by utilizing fuzzy logic which allows elements to have different levels of membership to classes and not to simply be associated with one single class. That is to say, it allows for some vagueness in categorizing some data. The schematic of a fuzzy system, how input data are *fuzzified*, is shown in Fig. 1. In this example, the element has a degree, or grade, of membership of 0.2 in *Low*, 0.8 in *Medium*, and 0 in *High*.

More formally, a fuzzy set is a pair $(X,\alpha )$ where *X* is a set and $\alpha$ is a membership function. This membership function $\alpha$ maps all elements of *X* to a number *between* 0 and 1

1 $$\begin{aligned} \alpha : X \rightarrow [0,1]. \end{aligned}$$

This is in contrast to traditional sets, which have functions which map each element of a set *X* to *either* 0 or 1.

The membership function, $\alpha$, which maps the set *X* to the fuzzy set *A* is written as $\mu _A$.

The membership function gives the degree of *similarity* of an element to a fuzzy set. This could also be stated as the distance between an element and a characteristic element of that set. It should be noted that this is not to be confused with *probability* of membership.

FPTs were first introduced, independent of each other, by Huang et al. [19] and Yi et al. [43] who called this type of model Fuzzy Operator Trees. The FPT model class is associated with other model classes, including fuzzy rule-based systems (FRBS) and fuzzy decision trees (FDT).

The goal of fuzzy logic was to allow the knowledge acquired from data to be expressed in a comprehensible form, very close to natural language, and mimic how an expert would convey their opinion. A doctor could describe their patient as either *young*, *middle-aged*, or *old*. Depending on the context *young* will have different boundaries, there is no quantitative boundary to describe the term. A 40-year-old person may be described as *young* if they have a heart attack but *old* if they have Chicken Pox. The degrees of *youngness* will also vary. Therefore, these terms, as well as terms like *tall*, *long*, and *hot*, are referred to as *fuzzy concepts*.

An advantage of using GE in the context of evolving structures for fuzzy rule base is the flexibility it gives in defining different partitioning geometries based on a chosen grammar [41].

The use of fuzzy sets allows for the creation of fuzzy rules. Similar to crisp rules, they yield an output based on a certain input or inputs. However, unlike crisp rules which state something is either *x* or *y* (e.g., true or false, positive or negative, etc.) fuzzy rules allow for degrees of truth to be incorporated into its statements. That is to say, something does not need to be entirely true or entirely false.

An example of a binary, crisp IF–THEN rule can be seen below:

- $$*$$ **IF** $${Cholesterol >140}$$
- $$*$$ **THEN** $${BMI > 25.0}$$.

The clear drawback of such a rule is the hard cut-off limit. If a person has a *Cholesterol* of 139.9, then the model will not predict that their *BMI* will be greater than 25, despite being having an almost identical *Cholesterol* to a person with 140.

An example of a fuzzy IF–THEN rule would be

- $*$ **IF** *Cholesterol HIGH*
- $*$ **THEN** *BMI HIGH*.

The fuzzy concepts of *Cholesterol HIGH* and *BMI HIGH* are described using fuzzy sets. These IF statements can combine many variables together:

- $*$ **IF** *Cholesterol HIGH* **AND** *Heart Rate HIGH*
- $*$ **THEN** *BMI HIGH*.

These combined rules can be used to identify relationships between input and output variables and create classifiers. The most popular of these classifiers are *fuzzy rule-based systems* (FRBS) and *fuzzy decision trees* (FDTs).

FDTs are an extension of standard *decision trees*. Despite their similar hierarchical structure, they are quite different that FPTs. They follow a top–down approach and work by continually partitioning the domain to build their single classifier.

FRBS are rule-based classifiers and are flat structures which use a fuzzy rule base to model the relationships in the data. Despite their obvious differences in representation (flat vs hierarchical), it has been shown that FPTs are a generalization of rule-based systems [36].

An FPT is a hierarchical, tree-like structure. The internal nodes are fuzzy logical and fuzzy arithmetic operators, and the leaf nodes are the fuzzified input variables and constants. Like traditional GP or GE trees, the information passes from the bottom of the tree to the top. An operator node takes the value or values of its descendants as inputs, performs the required operation, and conveys the result to its preceding node. Thus, an FPT implements a recursive mapping producing outputs in the [0,1] interval.

FPTs have independently been introduced by [19] and [43], who called this type of model Fuzzy Operator Trees. The FPT model class is related to several other model classes including fuzzy rule-based systems (FRBS) and fuzzy decision trees (FDT).

The following fuzzy operators are used, where *a* and *b* are the inputs to the operator:

2 $$\begin{aligned}&WTA = IF\{\}()..ELSE() \end{aligned}$$

3 $$\begin{aligned}&{\rm{MAX}} = {\rm{max}}(a, b) \end{aligned}$$

4 $$\begin{aligned}&{\rm{MIN}} = {\rm{min}}(a, b) \end{aligned}$$

5 $$\begin{aligned}&WA(k) = ka + (1 - k)b \end{aligned}$$

6 $$\begin{aligned}&{\rm{OWA}}(k) = k \cdot {\rm{max}}(a,b)+(1-k){\rm{min}}(a,b) \end{aligned}$$

7 $$\begin{aligned}&{\rm{CONCENTRATE}} = a^2 \end{aligned}$$

8 $$\begin{aligned}&{\rm{DILATE}} = a^{\frac{1}{2}} \end{aligned}$$

9 $$\begin{aligned}&{\rm{COMPLEMENT}} = 1 - a, \end{aligned}$$

where *WTA*, *WA* & *OWA* denote Winner takes all, Weighted Average, and Ordered Weighted Average, respectively. In the case of the *WA* and *OWA* operators, *k* will be a value created randomly within the range [0, 1]. Only one input will be provided in the case of the concentration, dilation, and complement. *WTA* will be the root node of every fuzzy tree. This function receives the score from each FPT and labels the individual corresponding to the highest scoring tree.

**Fig. 2:**
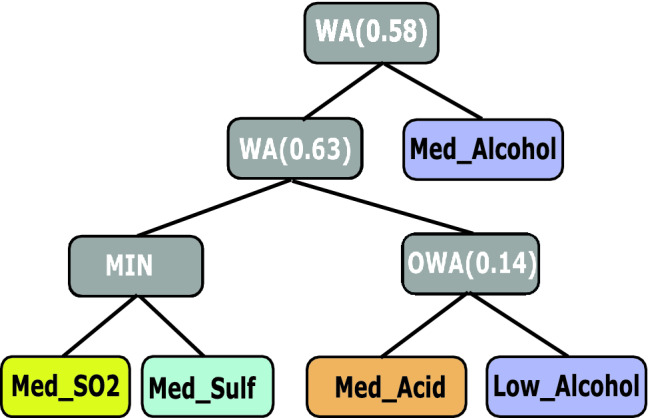
Tree representing the interpretable class “Good Quality Wine”, showing each variable with different color, taken from [25]

Figure 2 shows an example of an FPT, which was trained from a wine quality dataset, which contains various chemical properties of wines and an output of the wine’s quality. This FPT represents the fuzzy concept—a fuzzy criterion for—wine with a high quality.

To interpret a whole tree and grasp the fuzzy pattern it depicts, we first start at its root node. It represents the final aggregation (a simple average in this case) and outputs the overall evaluation of the tree for a given instance (a wine). Then, we proceed to its children and so forth. An interpretation of this tree could be:

*A high-quality wine fulfills two criteria. We call these two criteria—the left and right subtrees of the root node—criterion I and criterion II. Criterion I is fulfilled if the alcohol concentration of the wine is high or its density is high. Criterion II is fulfilled, if the wine has a high concentration of sulfates or a third criterion (III) is met. This is the case if both alcohol concentration and the wine’s acidity is low.*

FPTs were created with an emphasis on the representation of knowledge through a tree-shaped expression rather than representing it in the form of rules.

Hierarchical representation minimizes existing problems in rule-based systems, such as exponential increase in the number of rules with increasing entries and loss of interpretability when a large number of rules are required to achieve accuracy requirements. The tree is represented as a graph, favoring the human ability to recognize visual patterns, allowing the discovery of connections between the input variables and a class. These connections can be complicated to make when using models with a fixed set of rules.

To give a better interpretability to the evolved models, fuzzy logic is used to build more meaningful trees. To this end, it uses the following three linguistic terms for fuzzy labels: low, medium, and high (see Fig. 1).

To obtain a classifier one tree is created for each class, the classifier decision occurs in favor of the tree (class) that has the highest output value. Also, since each tree is considered a “logical description” of the class, it allows a more specific interpretation of the learning problem [37].

FPTs provide an alternative for the construction of accurate and interpretable fuzzy models. The interpretability that FPTs evolved using GE can offer has already been empirically shown [27].

#### Top–Down Induction of Pattern Trees

A successful method to create FPTs is seen in [37]. They called their approach Pattern Tree Top–Down Epsilon (PTTDE), a beam search technique. Epsilon determines the improvement required to continue to grow the tree.

The Beam Search learning scheme is quite “greedy”. This prevents optimal exploration of the search space and greatly increases the likelihood of the algorithm getting trapped in a local optimum. It also suffers from the *curse of dimensionality*. That is to say, if there is a large quantity of input features and the width of the beam is large, then the algorithm will use a lot of resources and time to evaluate all the possibilities. This leads to an exponential increase in the number of possible combinations.

### Cartesian GP

Genetic Programming (GP) examines the automatic generation of computer programs, inspired by the theory of evolution. The initial representation of GP was in a tree from [21]. CGP [24] is a flavor of GP with approximately 20 years of interesting and varied research works addressing a wide range of problem domains.

CGP uses graphs to represent solutions. Its distinguishing characteristic among other GP variants is its ability to encode computational structures as directed graphs using redundant genes. This redundancy serves CGP to get a very adaptable representation by allowing the outputs nodes to either connect or disconnect to nodes from previous nodes in the directed graph.

The synthesis of FPTs by CGP was proposed by [34] and their results indicated that FPTs synthesized by CGP are competitive with other classifier algorithms while at the same time being smaller than those obtained in [37].

The synthesis of FPTs by CGP can also be found in [35]. The authors apply the improvements in CGP proposed by [16] and implemented the well-known NSGA-II strategy to deal with two conflicting objectives, namely, the accuracy and the size of the tree.

The underlying difference between traditional forms of Linear GP (LGP) and CGP and their restrictions in connectivity was investigated in [42].

The difference between graph-based LGP and CGP is the means with which they limit the feed-forward connectivity of their directed acyclic graphs. In particular, CGP restricts connectivity based on the levels-back parameter, while LGP’s connectivity is implicit and is under evolutionary control as a component of the genotype.

Experimentally, it has been shown that programs evolved using CGP do not exhibit bloat [40]. However, using CGP to evolve programs in an arbitrary language can be problematic.

### Grammatical Evolution

GE is often thought of as variant of GP. It differs in that the space of legal programs it can explore is described by a grammar [33] or Attribute Grammar (AG) [31] given in Backus–Naur Form (BNF) . Crucially, it can evolve computer programs or arbitrary structures which can be defined using these grammars [3, 4, 39].

**Fig. 3:**
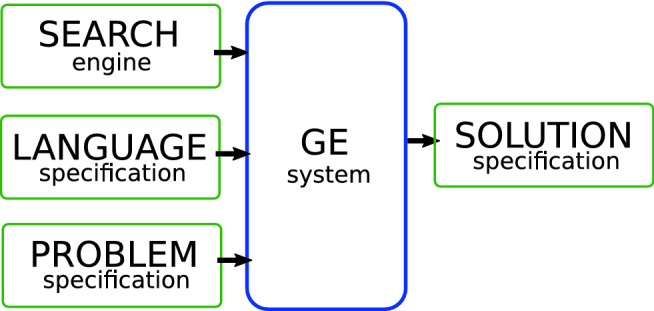
The GE system uses a search engine (typically a GA) to generate solutions for a given problem, by recombining the genetic material (genotype) and mapped onto programs (phenotype) according to a language specification (interpreter/compiler). Taken from [25]

The interchangeable design behind GE, as shown in Fig. 3, means that GE is very flexible. Any search techniques may be used, such as Simulated Annealing or Particle Swarm, but usually a variable-length Genetic Algorithm (GA) is employed to evolve a population of binary strings. A mapper is used to transform the strings onto a program using the grammar. Any program/algorithm can be used to evaluate those individuals.

**Fig. 4:**
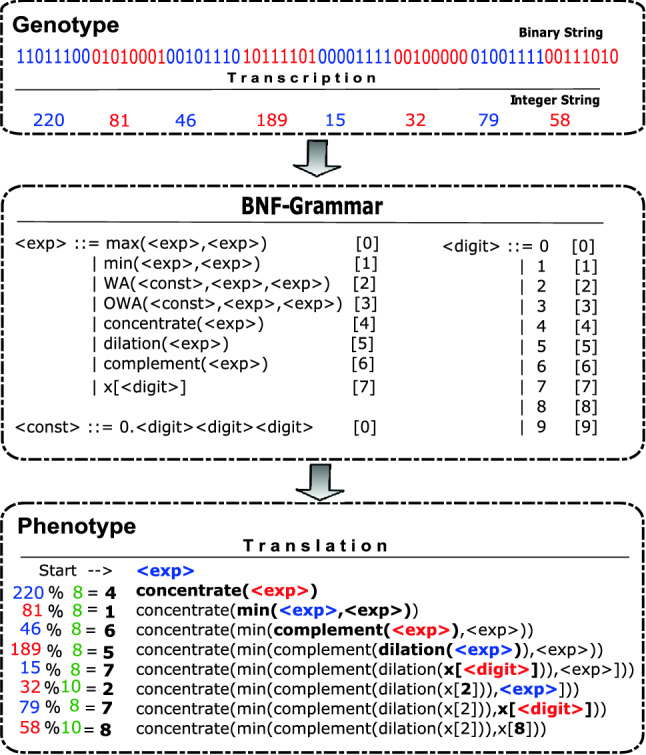
Example of a GE genotype–phenotype mapping process for the Iris dataset, where the binary genotype is grouped into codons (e.g., 8 bits; red & blue), transcribed into an integer string, then used to select production rules from a predefined grammar (BNF-Grammar), and finally translated into a sequence of rules to build a classifier (phenotype). Taken from [25]

The linear representation of the genome allows the use of the typical genetic operators of crossover and mutation in the manner of a typical GA. This is in contrast to tree-based GP. We use GE for our system, because its use of a grammar facilitates the adding of extra types in later versions, although GP could equally have been used in this case. Each individual’s chromosome contains in its codons (typically groups of 8 bits) the information necessary to select and apply the grammar production rules, constructing the final program which is evaluated. The mapping process is illustrated in Fig. 4.

Production rules for each non-terminal are indexed starting from 0. The mapping starts with the left-most non-terminal. To select the next production rule, the next codon value in the genome is read and interpreted using the formula: $p = c\; \%\; r$, where *c* represents the current codon value, $\%$ represents the modulus operator, and *r* is the number of production rules for the left-most non-terminal.

If the algorithm reaches the end of the genome, a wrapping operator is invoked. This continues the mapping process by returning to the start of the individual and reading the codons from the beginning of the genome. The mapping process stops when all of the non-terminal symbols have been replaced with terminal symbols, resulting in a valid program. If non-terminal symbols remain after a maximum number of iterations, the program is considered invalid and is penalized by being given the lowest possible fitness.

## Fuzzy GE

This section introduces Fuzzy GE, an evolutionary approach to generate classifiers with linguistic labels. The aim is to create meaningful models applied to binary and multi-classification problems.

### Standard Classification

There are many approaches used to evolve classifiers [12] and GE has been shown to be well suited for such a task [30]. One of the most popular methods for evolving a GP binary classifier is *Static Range Selection* (SRS) [22]. For multi-class classification, *Centred Dynamic Class Boundary Determination* (CDCBD) was proposed [45].

In binary classification, an input $\mathbf {x} \in \Re ^n$ has to be classified as belonging to one of two classes, $\omega _1$ or $\omega _2$. In this method, the goal is to evolve a mapping $g(\mathbf {x}):\Re ^n \rightarrow \Re$. The classification rule ${\mathcal {R}}$ states that pattern $\mathbf {x}$ is labeled as belonging to class $\omega _1$ if $g(\mathbf {x}) > r$, and belongs to $\omega _2$ otherwise, where *r* is the decision boundary value.

**Fig. 5:**
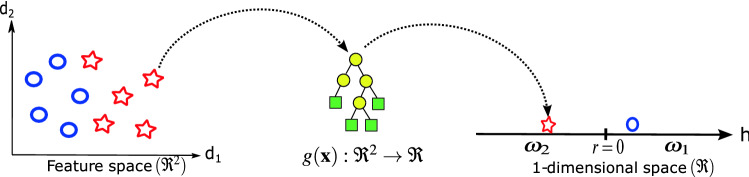
Pictorial representation of static range selection. An individual is given to the model which outputs a value. If this output exceeds the threshold value, it is classed as belonging to on class; if it is less than the threshold value, then it is classes as the other. In the example, the output of the tree is less than 0, the designated threshold, and is classed as belonging to $\omega _2$

The fitness function is defined to maximize the total classification accuracy after ${\mathcal {R}}$ is applied, normally setting the decision boundary to $r=0$. A data sample is passed to the tree which yields a score. If the score is below the boundary, it is labeled a particular class, and likewise, it is labeled the other class if it is above the boundary. This process is illustrated in Fig. 5.

The process for CDCBD is similar, with two or more boundaries existing, which can dynamically change to class each individual.

Both approaches only evolve one tree (or mapping) regardless of the number of classes and attempt to classify the individual based on its output from that tree. There are drawbacks to this approach as much effort needs to be expended into designing or hand-crafting class boundaries or creating systems to optimise them for each individual [13], which becomes increasingly more difficult as the number of classes increases.

### FGE Classification

An FPT classifier requires that one FPT be evolved per class in the problem. Evolving multiple trees simultaneously adds a great deal of complexity to the problem. In general, care must be taken and special operators, particularly when using crossover, must be created [2]. However, due to the separation between the search space and program space in GE and the grammar we have specified, it is not necessary to create any special operators in our approach, Fuzzy Grammatical Evolution (FGE).

**Fig. 6:**
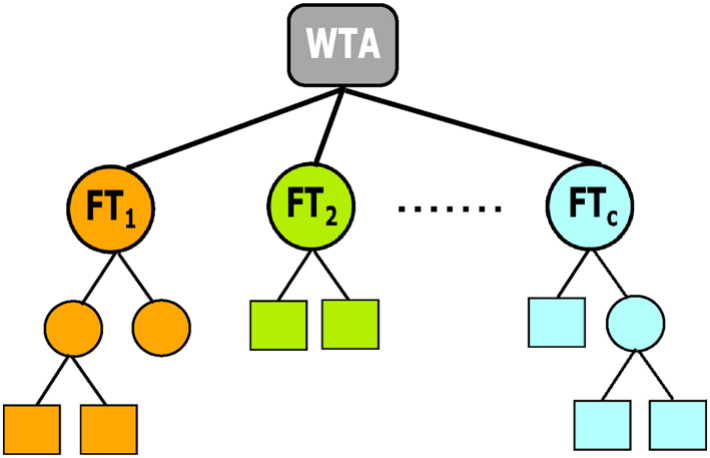
Pictorial representation of a multi-classifier evolved by Fuzzy Grammatical Evolution. This example has *c* classes, so *c* Fuzzy Pattern Trees are created, FT$_1$ to FT$_c$. Here, every Fuzzy Pattern Tree is given a different colour. FT$_1$ is the Fuzzy Pattern Tree concerning class 1, FT$_2$ is the Fuzzy Pattern Tree concerning class 2, and so on. The winner take all (WTA) function is at the root of the tree. This function assigns the individual to the class corresponding to the highest output of FT$_1$ to FT$_c$. For instance, if a problem had 3 classes, we need to create 3 Fuzzy Pattern Trees, FT$_1$, FT$_2$, and FT$_3$. If FT$_1$ has an output of 0.2, FT$_2$ an output of 0.6, and FT$_3$ an output of 0.3 for a particular instance, then the WTA function will assign this instance as class 2, as it has the highest score

The novel method involves evolving only one large solution. This solution comprises of FPTs, with each class having its own FPT, and a decision node at its root. Each FPT can therefore be thought of as a subtree of a larger classifier tree, as shown in Fig. 6, with the root node assigning the label to each individual. More formally, *i* mappings $f_i(\mathbf {x}):\Re ^n \rightarrow [0,1]$, where *i* is the number of classes in the problem to be evolved. The FPT, or subtree, ($f_1(\mathbf {x})$...$f_i(\mathbf {x})$) which confers the largest score to the individual is deemed the winner and the individual is labeled with the class that FPT represents.

**Fig. 7:**
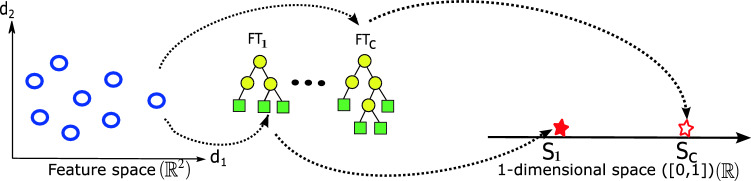
Graphical depiction of the mapping process from the feature space to a one-dimensional space [0,1] using a set of fuzzy trees $FT_1$ to $FT_c$

For example, if $f_1(\mathbf {x})$ yielded the largest score, the individual would be assigned to class 1. This is highlighted further in Fig. 7, where the second tree yields the better score, $S_c$, the hollow star. The individual is therefore assigned class *c*. This is in contrast to the methods described above which only produce 1 score per individual and assign it a label based on the score’s position relative to a boundary(s).

FGE does not require the use of any protected operators when evolving multiple trees due to the unique separation between genotype and phenotype, and only needs grammar augmentation to address different problem types.

### Fuzzy Representation

Fuzzy logic is used to give better interpretability to the evolved models and give a deeper meaning to the trees. It uses the following linguistic terms for fuzzy labels: low, medium, and high. The input space was partitioned uniformly to create fuzzy partitions.

The fuzzy operators used are described in “Fuzzy Sets and Fuzzy Pattern Trees”. The values of the inputs of the operated nodes are *a* and *b*. In the case of the Weighted Average (WA) and Ordered Weighted Average (OWA) operators, the value *k* is produced randomly the range (0, 1) exclusive. For concentration, dilation, and complement, only one input is needed. The Winner Takes All (WTA) operator will be the root node of every fuzzy tree. Each FPT gives its score to this function. It then classifies the individual corresponding to the largest scoring FPT.

The grammar can be changed and augmented in GE to include different operators or fuzzy terms to create different trees.

### Parsimony Pressure

Maximizing a model’s accuracy, or similarly minimizing its error, is the usual focus of research. However, the interpretability of these models has continued to grow in significance, with numerous workshops and conferences now devoted to the area [1]. For an FPT to be interpretable or comprehensible, and therefore allow it to serve as a description of the class, it is of utmost significance for the evolved solutions to remain as small as possible and avoid bloat in the final programs [12]. GP’s ability to find highly dimensional, highly non-linear solutions is praised and often thought of as its strong point. However, this often leads to a significant loss of interpretability. One of the main advantages CGP enjoys over standard GP, and other GP variants, is its inherent lack of bloat [40].

Parsimony pressure is an approach to limit the size of individuals [23]. It is not a GP-specific technique and can be deployed whenever arbitrarily sized representations are inclined to become far too large. It may generally be divided into two varieties: parametric and objective-based parsimony pressure.

Parametric parsimony pressure directly uses the size of the individual when calculating its fitness. Objective-based parsimony pressure considers the size of the individual as a separate objective to be considered in a multi-objective optimization procedure. A common example of two objectives is tness and size.

### Ensemble Techniques

While sacrificing the accuracy of a classifier to gain interpretability may be acceptable for most users of a model, the new interpretable models must remain highly competitive with the black-box one they are replacing. Ensembling offers an attractive approach to boost the performance of FPTs [9]. Generally, it combines the predictions of many “weak” classifiers to improve the overall performance. This would not harm the interpretability of the system providing each individual model remained small and the number of models which make up the ensemble also remains low. We considered three types of ensemble methods; aggregation, adaptive boosting, and gradient boosting.

*Aggregation* randomly partitions the data differently every run and produces a variety of learners. The outputs of these are combined together equally to yield a final prediction.

*Boosting* attempts to correct the mistakes of previous models and build new models which can search in the areas the previously trained models struggle to classify accurately.

*Adaptive boosting* creates a new training set by sampling with replacement on the original training set [14]. Data which have been incorrectly classified by the previous models trained will be far more likely to appear in the new training set than those points which have been correctly classified. Each model does not have an equal say, as is the case with aggregating. The model is given a greater say in the final decision if it is seen to classify the data accurately.

Finally, a *gradient boost* approach was investigated [15]. This method takes the errors from the previous models and attempts, step-by-step, to reduce the errors by introducing new weak learner. Due to an FPT consisting of *n* trees per class and the output of each tree being confined to [0,1], a traditional gradient boost approach would not be possible. A new approach is constructed by taking the output of each FPT and updating it by combing with the output of new FPT found and then scaling the final result to ensure the total score remains in [0,1], as shown in Eq. 17. This is repeated a number of times until the stopping criteria are met.

Aggregation has a distinct advantage in that it may be performed in parallel, whereas both adaptive and gradient boosting need to be performed sequentially.

Three sets of experiments were run in this paper. The first uses standard GE and the second implements an adjusted parsimony pressure to bias the selection by slightly punishing a solutions fitness as it grows in size. The size is defined as the maximum depth of any of the *n* FPTs in a particular solution. The final set of experiments investigated the various ensemble methods to try to enhance the classification performance of the FPTs. The ensemble methods were only performed on the binary classification problems. This was due to two reasons; any improvement given by boosting would be easier to identify in binary problems and the added complexity required for multi-class classification.

## Experimental Setup

The approach was compared with a diverse set of state-of-the-art classification algorithms and was also compared with one other FPT related method, FPTs evolved using CGP (FCGP) [34]. The benchmark classification problems used in [34] were used to produce a fair comparison between the two approaches. The full experimental setup for CGP and each of the other benchmark classification techniques which FGE is compared against can be seen in [34]. Table 3 shows the results.

The experiments were run on Intel Xeon Gold 6138 2.00GHz CPU. LibGE, due to appear online shortly, was used for evolution and statistical analysis was done using R. It should be noted that the current implementation of LibGE is only single threaded.

### Datasets

Eight benchmark datasets were chosen to run the experiments on, all of which can be found in the UCI and CMU repositories online [11, 38]. Six binary classification problems and two multi-class problems are considered. The size of each dataset, as well as the number of classes and variables, are displayed in Table 1.

**Table 1:** Benchmark datasets for binary and multi-class classification problems, taken from the UCI repository and and the CMU repository

Datasets	Short	#Classes	#Variables	#Instances
Binary				
Lupus	Lupus	2	3	87
Haberman	Haber	2	3	306
Lawsuit	Law	2	4	264
Transfusion	Transf	2	4	748
Pima	Pima	2	8	768
Australian	Austr	2	14	690
Multiclass				
Iris	Iris	3	4	150
Wine	Wine	3	13	178

### GE Parameters

The experiments were run for 50 generations with a population size of 500. Sensible Initialisation and effective crossover were used [32]. Fivefold cross-validation was used. This was repeated 5 times for a total of 25 runs.

The ensemble methods were performed with a modified set of parameters.

For aggregation, the data were randomly split at the beginning of each run 40% for training, 35% for validation, and an identical 25% for testing. This is similar to the approach of random forest with the exception being that the training sample was selected without replacement from the pool of individuals. The best seven performing models on the validation set are chosen and used for aggregation for the test data.

For adaptive boosting and gradient boosting, the data were split 75% training and 25% for test. This was repeated at the beginning of each run, so that a different, randomized training and test data were used in each experiment. There were ten boosting iterations and the total cost of each run remained the same. That is to say, every 5 generations, the best individual was added to the ensemble and the weights of each sample updated and the evolution was restarted. This yielded the full ensemble after 50 generations.

**Table 2:** List of the main parameters used to run GE

Parameter	Value
Folds	5
Runs	25 (5 per fold)
Total Generations	50
Population	500
Replacement	Tournament
Crossover	0.9 (Effective)
Mutation	0.01
Initialisation	Sensible
*For Adaptive and Gradient Boosting:*	
Boosting Iterations	10

These experimental values lead to a larger computational cost than the CGP experiments [34]. The full experimental setup can be seen in Table 2. Each run took approximately 90 min to complete.

**Fig. 8:**
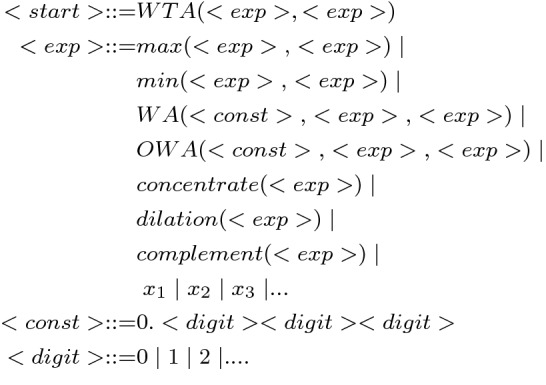
Grammar used to evolve a Fuzzy Pattern Tree for a binary dataset. The *WTA* node can be augmented by adding extra $<exp>$ to include as many subtrees as necessary, making it a multi-class grammar

The binary classification grammar used in experiments can be seen in Fig. 8. The *WTA* node contains two $<exp>$ non-terminals which need to be expanded. These will be the FPTs for each class when they are fully expanded. Two FPTs are required for binary classification. To make this grammar suitable for multi-class classification, it needs to be augmented by simply adding more $<exp>$ symbols in the expression. Three classes require three $<exp>$ symbols and so on. Constants were created using the standard GE approach of digit concatenation [6].

### Fitness Function

The fitness function chosen seeks to minimize the RMSE for each individual, similar to the approach used previously in CGP evolution. This is seen in Eq. 11. The benchmark datasets used in experimentation are quite balanced and it was decided a non-standard fitness function, such as cross entropy, was not required. However, it may be necessary in future experiments to alter this if the data are very unbalanced

10 $$\begin{aligned} {\rm{RMSE}}= & {} \sqrt{\sum _{i=1}^{n} \frac{(\hat{y_i} - y_i)^2}{n}} \end{aligned}$$

11 $$\begin{aligned} F= & {} 1 - {\rm{RMSE}}. \end{aligned}$$

The fitness function for FGE with lexicographic parsimony pressure which aims to find small, interpretable solutions is calculated by penalizing the solution as its size grows. It is computed as follows:

12 $$\begin{aligned} F_{L} = 1 - {\rm{RMSE}} \times 0.99 - {\rm{MaxDepth}} \times 0.01. \end{aligned}$$

Experiments using fitness function *F* are denoted as *FGE* in the results section. Experiments which used $F_L$ are signified by $FGE-L$

The max depth of each FPT in an individual is averaged to find the mean depth of a solution. For a binary classifier *C* with two FPTs, $FPT_{1}$ and $FPT_{2}$, the mean depth would be

13 $$\begin{aligned} \begin{array}{l} {\rm{MeanDepth}}(C) = \frac{1}{2} \left( {\rm{MaxDepth}}(FPT_{1}) + {\rm{MaxDepth}}(FPT_{2}) \right) . \end{array} \end{aligned}$$

For adaptive boosting, the influence each classification model has in the final classification is calculated as

14 $$\begin{aligned} \alpha _i = \frac{1}{2}ln \left( \frac{1-{\rm{Error}}_i}{{\rm{Error}}_i} \right) . \end{aligned}$$

The gradient boost and aggregation experiments used fitness function 12. A larger penalty was applied in the adaptive boosting experiments, equation 15, to produce very small trees to add to the forest, which are preferred in adaptive boosting [14]. The weight of a sample *j*, $w_j$, after evolution of $i+1$ FPTs was updated per equation 16.

15 $$\begin{aligned} F_{Ada}= & {} 1 - {\rm{RMSE}} \times 0.99 - {\rm{MaxDepth}} \times 0.05 \end{aligned}$$

16 $$\begin{aligned} w_{j}^{i+1}= & {} \frac{1}{ z_{i+1} } \times {\left\{ \begin{array}{ll} w_{j}^{i}\times e^{-\alpha _{i+1}} &{} \text {if classified correctly by classifier} i+1\\ w_{j}^{i}\times e^{\alpha _{i+1}} &{} \text {otherwise}. \end{array}\right. } \end{aligned}$$

Here, $z_{i+1}$ is a normalization term to ensure all the weights sum to 1 and $\alpha _{i+1}$ is calculated as in 14.

For gradient boosting, a new FPT is added to an ensemble of $i-1$ trees for class *c* as

17 $$\begin{aligned} S^{c}_i(x) = \frac{S^{c}_{i-1}(x) + 0.1\times FPT ^{c}_i(x)}{1.1}, \end{aligned}$$

where $S^{c}_i$ is the score for the ensemble of class *c* consisting of *i* FPTs, $S^{c}_{i-1}$ is the score at the previous iteration, and ${FPT}^{c}_i(x)$ is the output of the FPT at iteration *i* to be added to the ensemble.

## Results

The experimental results are shown in Table 3. The mean test performance from all 25 runs of FGE is presented. Other methods performances are taken from [35]. The best result for each problem is highlighted in bold. The binary problems (1-6) are shown first, with the multi-classification problems (7-8) following after.

The first column in Table 3 shows the results for FGE. The second shows FGE with lexicographic pressure applied. The third, fourth, and fifth columns show the results for CGP (FCGP), Support Vector Machine with Linear Kernel (SVM-L), and Random Forest (RF), respectively. The sixth column shows Support Vector Machine with Radial Basis Function Kernel (SVM-R), and finally, column seven shows the Pattern Tree Top–Down Epsilon (PTTDE). This was the original proposed technique used to produce FPTs [37]. For those experiments, epsilon was set to 0.25%. Epsilon determines the improvement required to continue to grow the tree.

**Table 3:** Classification performance comparison of FGE versions against previous related work results, showing average classification on the test data for the best solution found per run

Dataset	FGE	FGE-L	FCGP	SVM-L	RF	SVM-R	PTTDE
Lupus	0.73	0.73	0.74	0.74	0.62	0.73	**0.77**
Haber	**0.74**	0.72	0.73	0.72	0.65	0.71	**0.74**
Law	0.96	0.94	0.93	**0.99**	0.97	0.96	0.94
Transf	0.76	**0.77**	0.76	0.76	0.70	0.73	**0.77**
Pima	0.74	0.74	0.72	**0.77**	**0.77**	0.71	0.76
Austr	**0.86**	**0.86**	0.85	**0.86**	0.79	0.85	0.85
Iris	**0.96**	**0.96**	0.95	**0.96**	0.95	0.95	0.95
Wine	0.83	0.83	0.90	**0.98**	**0.98**	**0.98**	**0.98**

Bold indicates the best-performing method

A Friedman test was performed to compare the performance of the classifiers. This test showed that no evidence there was one classifier that was statistically significantly better than all others.

It can be seen that FGE obtained very competitive performance compared with the previous experiments. However, it was noticeably outperformed in one benchmark, Wine, however. In this benchmark, it was the worst-performing classifier of all considered. FGE achieved mean best performance of 83%, compared to 98% found by SVM, RF, and PTTDE. It was also noticeably worse than the result achieved by CGP, which reached 90%. The reason for FGE’s inconsistent performance on the non-binary problems requires further investigation. It is possible the small size and the imbalance of the wine dataset resulted in FGE’s poor performance.

FGE attained the best performance in three problems: (i) Haberman—FGE achieves 74%, outperforming all and matching PTTDE as the best result; (ii) Australian—FGE and FGE-L score 86% for a tie in best-performing classifier; and (iii) Iris—FGE and FGE-L again equal the best-performing classifier attaining 96%. Intriguingly, FGE-L achieves best in class performance, 77%, on the Transfusion problem.

As well as these results, FGE and FGE-L both reach competitive performance on the remaining classification problems, with the only outlier being the Lupus dataset. On the Lupus problem, FGE reaches similar performance as FCGP, 73% and 74%, respectively, but PTTDE produced the best accuracy, 77%. FGE-L performs equivalently, finding 73% accuracy.

FGE-L can be seen to produce much smaller solutions than those found by FGE, as shown in Table 4. “SizeReduc” represents the reduction of the average tree size found by FGE-L compared to FGE. Note that if an FPT contains just a leaf node, it will be given a depth of 0.

FGE-L was able to discover remarkably smaller trees across all the problems considered. Specifically, the Haberman, Pima, and Australian problems were all seen to have a reduction in size by over 80%. Crucially, parsimony pressure does not appear to greatly affect the performance. A minor decrease in accuracy is seen in two problems: Haberman and Lawsuit. FGE is statistically significantly better on the Haberman dataset, with no difference seen between FGE and FGE-L in any other benchmark. Strikingly, there was an increase in the performance on the Transfusion problem by 1%. This major reduction may point to bloat being a significant problem in FGE. A pressure of 1% of size was applied in these experiments but tuning this parameter and how it is best applied is an avenue for future research.

FGE-L was the best-performing classifier on the Transfusion, Iris, and Australian problems. On the problems considered, there seems to be very little, and oftentimes none at all, trade-off in performance associated with evolving smaller trees. It is possibly the case that the global optima for these problems were smaller trees. This requires further investigation on larger, more complex problems. However, the results hint strongly that bloat may be an issue in FGE.

FGE was statistically significantly better than FCGP on the Haberman, Lawsuit, Pima, and Australian benchmarks. FGE-L was significantly better than FCGP on the Transfusion and Australian problems. FCGP significantly outperformed both FGE and FGE-L on the Wine dataset.

**Table 4:** Average size comparison in terms of the tree depth between fuzzy pattern trees approaches; FCGP, FGE, and FGE-L

Dataset	FCGP	FGE	FGE-L	SizeReduc
Lupus	**1.65**	7.84	2.38	70%
Haber	1.85	9.42	**0.2**	98%
Law	1.05	5.02	**0.98**	79%
Transf	2	6.76	**1.54**	77%
Pima	**1**	6.7	**1**	85%
Austr	1.5	5.12	**0.92**	82%
Iris	1.24	1.8	**0.64**	64%
Wine	1	2.47	**0.68**	72%

Best results are in bold. SizeReduc is the average size reduction seen in FGE-L vs FGE

CGP was previously seen to produce much smaller trees than those using PTTDE and will be used to compare to the trees found using GE [35]. The trees found using CGP are much smaller than those found using FGE but larger than those using FGE-L. When the search is biased towards smaller sized individuals, FGE-L finds smaller solutions in seven problems. Due to these small sizes, FPTs found using FGE-L should lead to very interpretable results.

On the benchmarks considered, SVM-L was seen to be the best performing method, attaining best performance on 5 of the the benchmark problems. On the other hand, SVM-L does not allow any interpretability of its solutions. FGE was best performing on three problems, FGE-L was best performing on three problems and FCGP was not seen to be best on any. FGE beat or equalled FCGP in 6/8 problems studied and FGE-L evolved the smallest trees in all but one problem, Lupus, but was able to beat FCGP in 5/8 problems.

The mean size of the final trees found by FGE, FGE-L with parsimony pressure, and CGP are shown in Table 4, and best results are in bold.

**Table 5:** Best-performing individual found for each ensemble method compared to best individual found using regular FGE

Dataset	FGE	Aggr’n	AdaBoost	GradBoost
Lupus	**0.94**	0.50	0.86	0.86
Haber	**0.82**	0.67	0.80	0.76
Law	0.98	0.96	**0.99**	0.97
Transf	**0.78**	0.77	0.76	0.77
Pima	**0.77**	0.75	**0.77**	0.76
Austr	0.88	0.87	0.87	**0.90**

Best-performing method is shown in bold

The ensemble methods were seen to have mixed performance on the problems considered. The results can be seen in Table 5, FGE had the best performance on the Lupus and Haberman datasets. This was likely due to the very small dataset each of the problems have, meaning that the ensembled classifiers may be prone to overfit. This is a particular concern for the aggregation approach, with only 40% of the dataset used to train the model. Gradient boosting found the best-performing model for the Australian model, besting FGE by over 2%. AdaBoost was best on Lawsuit, 99%, and tied for best on Pima with FGE, achieving 77%. FGE was best on the Transfusion dataset, attaining 78%. Aggregating was not seen to perform best on any benchmark considered, being noticeably outperformed on the Transfusion, 76% vs 78%, and Australian, 87% vs 90%.

While boosting was seen to match or improve classification performance of FGE in three examples, without any further computational cost, it was seen to overfit badly on the Lupus dataset. It remains to be investigated what loss of interpretability comes from combing many FPTs to form an ensemble against considering just one FPT. Due to the very similar performance between standard FGE and the ensemble methods, any loss of interpretabilty associated with their implementation may not be justified.

## Conclusions

This paper proposes a new approach to evolving Fuzzy Pattern Trees. It uses Grammatical Evolution as the learning algorithm, an approach we call Fuzzy Grammatical Evolution. The experimental results showed that Fuzzy Grammatical Evolution has comparable performance in classification accuracy with some of the best classification algorithms available. Grammatical Evolution was seen to exceed the performance of Cartesian Genetic Programming on the problems considered.

Fuzzy Grammatical Evolution was seen to exhibit inconsistent performance on the multi-class classification problems, achieving best results on the Iris problem and achieving worst results on the Wine benchmark. For problems requiring many Fuzzy Pattern Trees, it may improve performance to evolve each Fuzzy Pattern Tree individually and not evolve the ensemble of Fuzzy Pattern Trees all at once. This requires further investigation.

As interpretability is a key concern, experiments were conducted on methods to control program size. A simple pressure was applied by penalizing the fitness of individuals as they grew larger. We found that the final performance was unaffected by employing such measures and Fuzzy Grammatical Evolution with this pressure applied found both smaller and more accurate trees than those found using Cartesian Genetic Programming. This suggested that programs evolved using standard Fuzzy Grammatical Evolution are growing unnecessarily large and bloat was a considerable factor to consider in future experimentation.

In a final set of experiments, various ensemble techniques were investigated. This was in a bid to increase the accuracy of the final models where there may be a large discrepancy between them and black-box approaches. Adaptive boosting was seen to find better models than Fuzzy Grammatical Evolution on less than half the benchmarks considered. Further works must be done on larger, real-world datasets with more than three classes to fully gauge the improvements boosting may give over standard Fuzzy Grammatical Evolution.

Our experimentation verified that Fuzzy Pattern Trees are a viable replacement of the classic rule-based fuzzy models. This is due to their hierarchical structure which yields a more compact representation and allows for a better compromise between the accuracy and the simplicity of the model. Importantly, it also gives an interpretable model. That is to say, the knowledge which has been obtained in the learning process can be extracted from the final model and is shown to a user in understandable terms.

Several avenues of future research can be explored from the present work. The proposed algorithms should be evaluated on other machine learning problems, such as unsupervised clustering.

Another interesting course of action is to try other techniques to reduce the size of the trees, such as regularization or encapsulation [28], or hybridise GE with another technique to optimise smaller models already found [29]. Furthermore, the use of different types or sets of grammar could be investigated.

A final potential route for future exploration is to empirically examine if smaller tree sizes do offer more interpretability to the user. Some work on validating the interpretability of the final solutions found has already been carried out [26]. It is possible that some other metric, the number of variables used or the presence/absence of particular subtrees for example, may grant better interpretability. This must be examined.
